# Supplementary material for: Concave Ni(OH)2 Nanocube Synthesis and Its Application in High-Performance Hybrid Capacitors
Source: Nanomaterials (Basel). 2023 Sep 11;13(18):2538. doi: 10.3390/nano13182538 (PMC10537329; doi:10.3390/nano13182538)
Supplement: Supplementary file 1 [file nanomaterials-13-02538-s001.zip › nanomaterials-2579892-supplementary.pdf]

Supporting Information

# Concave $Ni(OH)_2$ Nanocube Synthesis and Its Application in High-Performance Hybrid Capacitors

**Nan Cong [1], Pan Li [2], Xuyun Guo [3] and Xiaojuan Chen [1,*]**

[1] Beijing Academy of Quantum Information Sciences, Beijing 100193, China
[2] Institute of Analysis and Testing, Beijing Academy of Science and Technology, Beijing 100089, China
[3] Centre for Research on Adaptive Nanostructures and Nanodevices (CRANN) & Advanced Materials Bio-Engineering Research Centre (AMBER), School of Chemistry, Trinity College Dublin, D02PN40 Dublin, Ireland
* Correspondence: chenxj@baqis.ac.cn

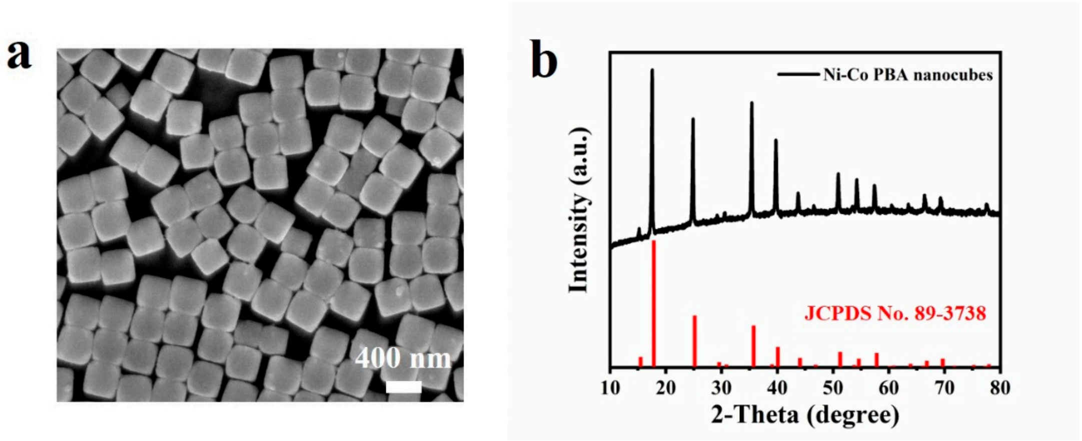


Figure S1. (a) SEM image and (b)XRD pattern of Ni-Co PBAs nanocubes with a size of 400 nm.

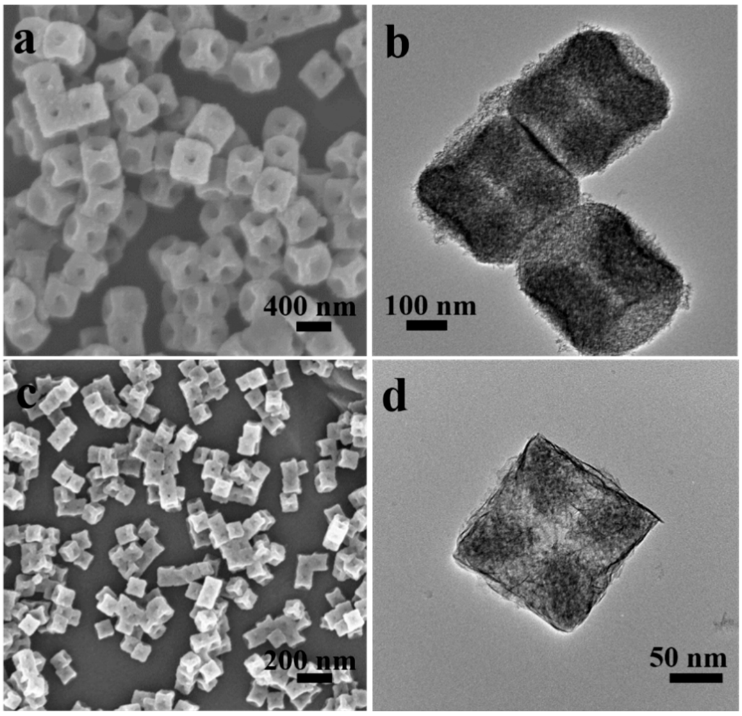


Figure S2. SEM images and TEM images of concave $Ni(OH)_2$ nanocubes with average size of (a,b) 350 nm and (c,d) 100 nm, respectively.

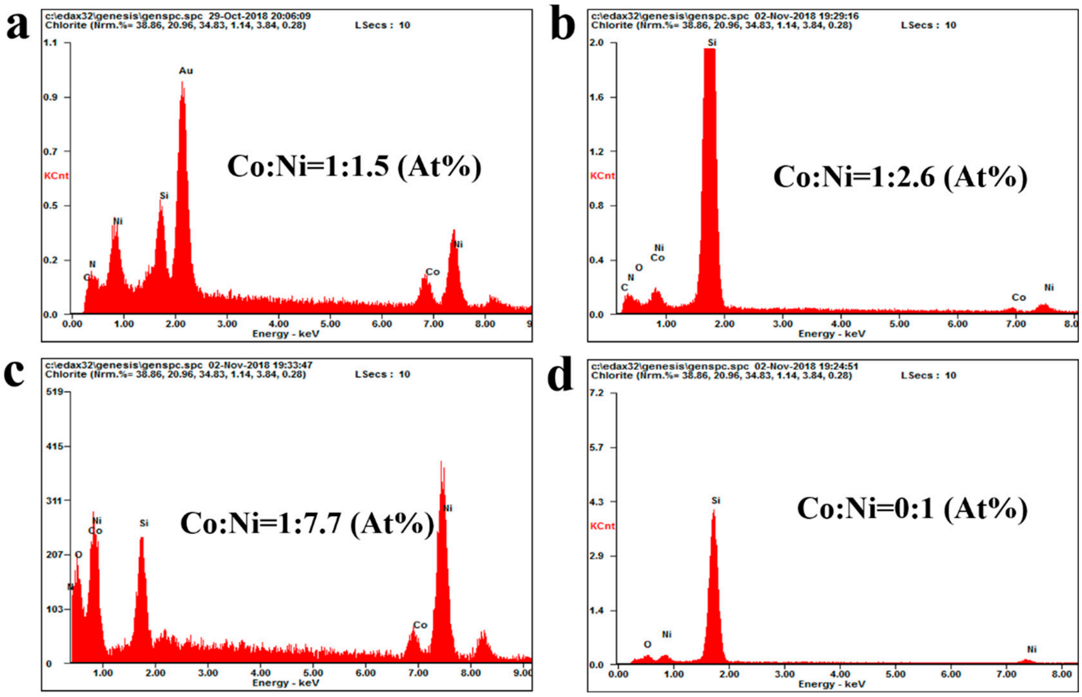


Figure S3. EDS spectra of the as-obtained products after reaction for 0 h (a), 1 h (b), 3 h (c), and 6 h (d).

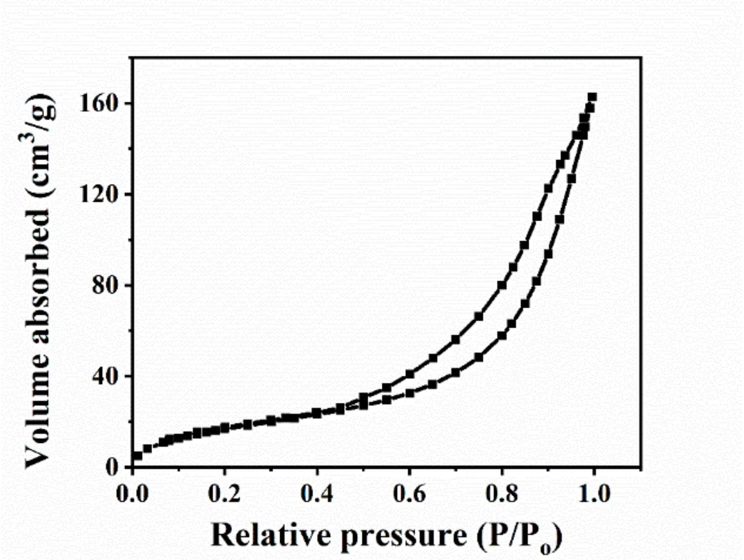


Figure S4. Nitrogen adsorption–desorption isotherms of the concave $Ni(OH)_2$ nanocubes.

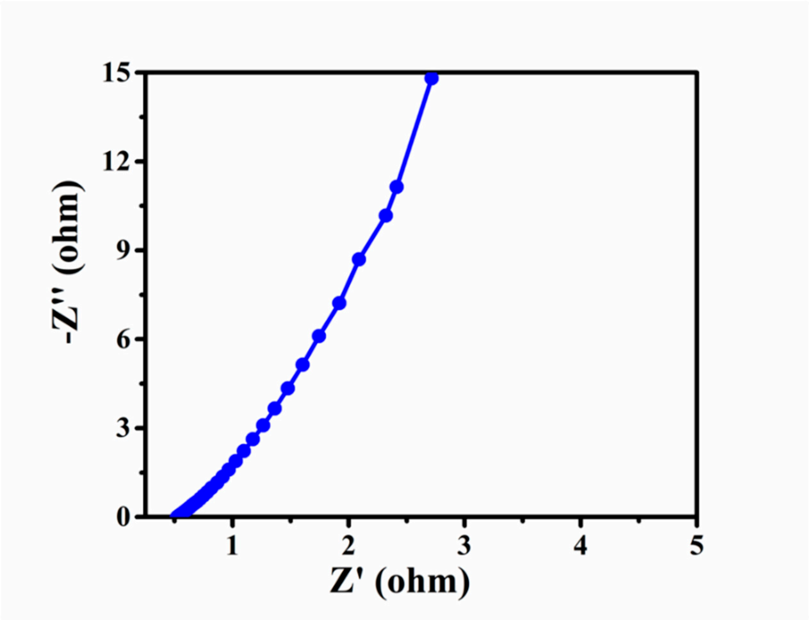


Figure S5. Nyquist plot of concave $Ni(OH)_2$ nanocubes.

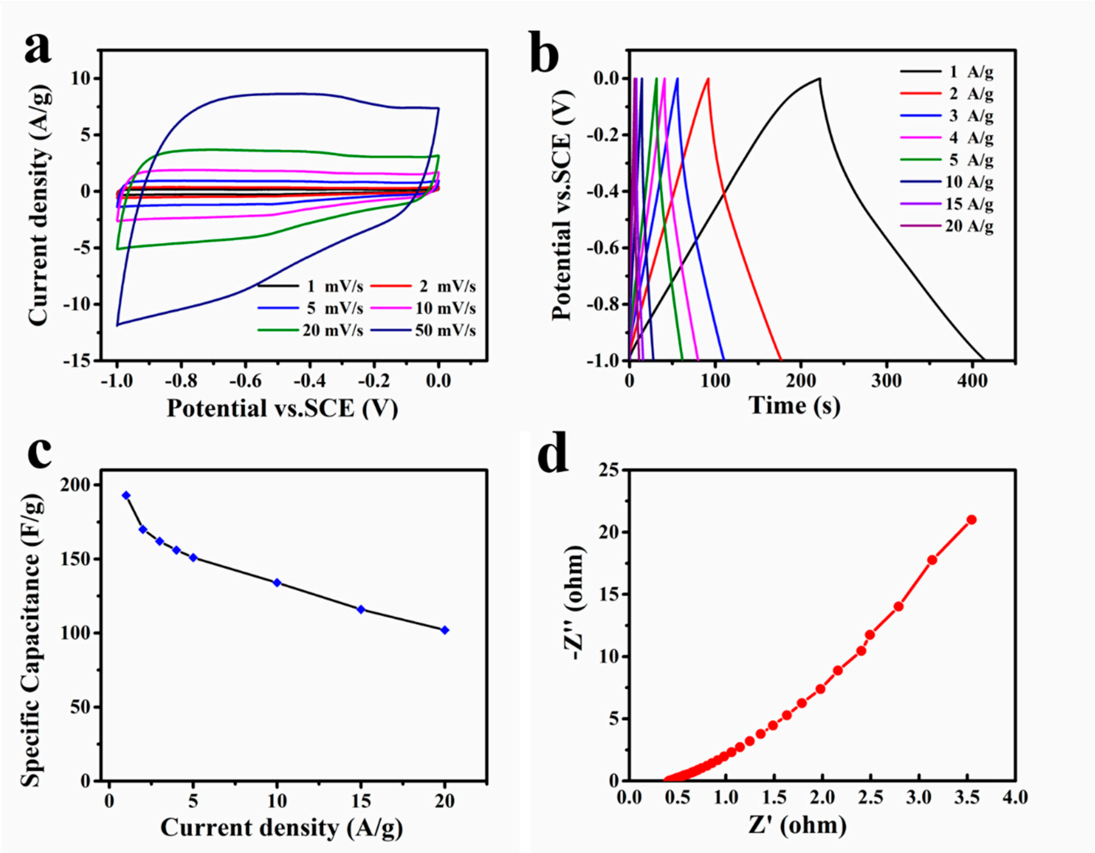


Figure S6. The electrochemistry properties of graphene: (a) CV curves at different scan rates, (b) Charge-discharge curves at varied current densities, (c) Specific

capacitances at a series of current densities, (d) Nyquist plot of graphene collected from the electrochemical impedance measurements.

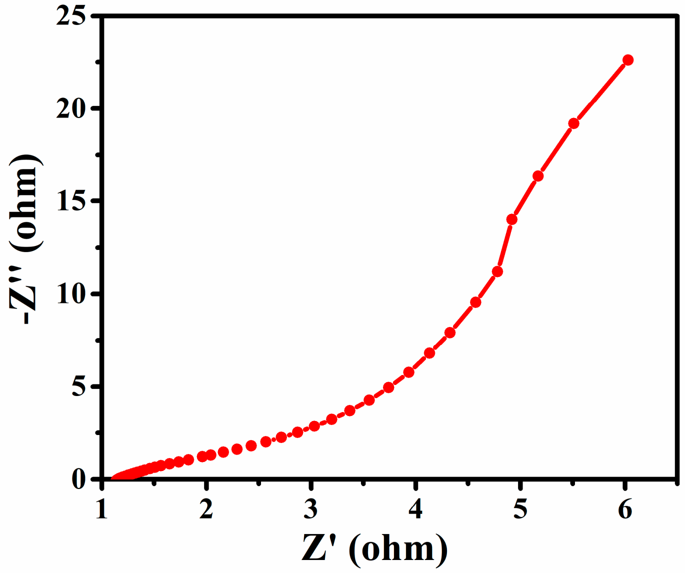


Figure S7. Nyquist plot for concave $Ni(OH)_2$ nanocubes//graphene hybrid capacitor.

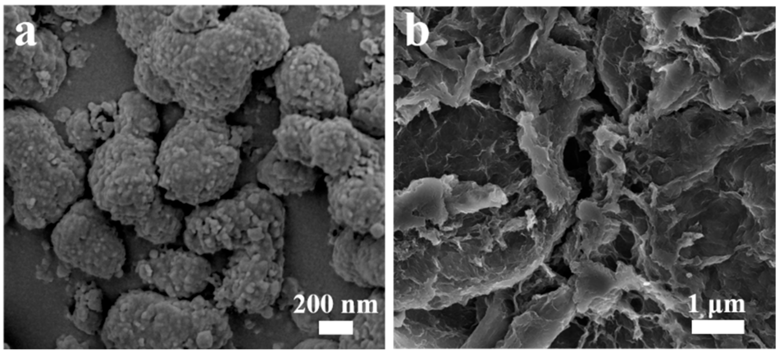


Figure S8. SEM image of concave $Ni(OH)_2$ nanocubes and graphene after 4000 cycles.
